# Upregulation of PDZK1 by* Calculus Bovis Sativus* May Play an Important Role in Restoring Biliary Transport Function in Intrahepatic Cholestasis

**DOI:** 10.1155/2017/1640187

**Published:** 2017-01-04

**Authors:** Dong Xiang, Tao Wu, Cheng-Yang Feng, Xi-Ping Li, Yan-Jiao Xu, Wen-Xi He, Kai Lei, Hong-Jiao Cai, Cheng-Liang Zhang, Dong Liu

**Affiliations:** ^1^Department of Pharmacy, Tongji Hospital, Tongji Medical College, Huazhong University of Science and Technology, Wuhan, China; ^2^Department of Pharmacy, Puai Hospital, Tongji Medical College, Huazhong University of Science and Technology, Wuhan, China

## Abstract

Intrahepatic cholestasis is a main cause of hepatic accumulation of bile acids leading to liver injury, fibrosis, and liver failure. Our previous studies proved that* Calculus Bovis Sativus *(CBS) can restore biliary transport function through upregulating the multidrug resistance-associated protein 2 (MRP2) and breast cancer resistance protein (BCRP) in 17*α*-ethynylestradiol- (EE-) induced intrahepatic cholestasis rats. The regulation mechanism of CBS on these transporters, however, remains unclear. This study was designed to evaluate the possible relationship between the effect of CBS on transport activities and the regulation of CBS on the expression of PDZK1, a mainly scaffold protein which can regulate MRP2 and BCRP. Intrahepatic cholestasis model was induced in rats with injection of EE for five consecutive days and then the biliary excretion rates and cumulative biliary excretions were measured. The mRNA and protein expression levels of PDZK1 were detected by reverse transcription-quantitative real-time polymerase chain reaction, western blot, and immunohistochemical analysis. When treated with CBS, cumulative biliary excretions and mRNA and protein expressions of PDZK1 were significantly increased in intrahepatic cholestasis rats. This study demonstrated that CBS exerted a beneficial effect on EE-induced intrahepatic cholestasis rats by restoring biliary transport function, which may result from the upregulation of PDZK1 expression.

## 1. Introduction

Intrahepatic cholestasis is a group of clinical syndromes that systemically and intrahepatically accumulates excessive toxic bile acids inducing hepatobiliary injury finally [1]. In human, intrahepatic cholestasis is one of the most common and devastating manifestations in many hereditary and acquired liver diseases, such as progressive familial intrahepatic cholestasis, hepatitis, drug-induced liver injury, intrahepatic cholestasis of pregnancy, and primary biliary cirrhosis [2]. At present, medical therapy can be attempted in a specific subgroup of patients [3], and drugs for treating cholestasis are quite limited. Ursodeoxycholic acid, as the only medicine approved by FDA to treat intrahepatic cholestasis [4], allows a normal life expectancy for about two-thirds of patients at the early stage, without additional therapies. Since the remaining patients cannot be controlled with ursodeoxycholic acid [5], finding new drugs that protect against intrahepatic cholestasis is of significant importance.

*Calculus Bovis* (Niuhuang), a traditional Chinese medicine derived from pigment gallstones of cow, has been widely used to relieve fever, to diminish inflammation, and to normalize gallbladder function for more than two thousand years in China [6]. Given that* Calculus Bovis* is insufficient of nature sources,* Calculus Bovis Sativus* (Tiwai Peiyu Niuhuang, CBS) was invented as the substitute in the medicinal preparations. Many studies have shown that CBS shares basically the same property, structure, composition, content, and clinical efficacy with those of* Calculus Bovis *[6–8]. Moreover, because of its complex components including bilirubin, cholic acid, bile acids and cholesterol, and so forth, we have established HPLC fingerprints for the effective quality control of CBS [8]. Our previous study indicated that CBS treatment had a beneficial effect on 17*α*-ethynylestradiol- (EE-) induced intrahepatic cholestasis rats and significantly increased the mRNA and protein expressions of multidrug resistance-associated protein 2 (MRP2) and breast cancer resistance protein (BCRP), both important proteins on the apical membrane of hepatocytes involving in the transport of bile acids, bilirubin, drugs, and so on [9]. But the possible regulatory mechanism of CBS on these transporters is still unclear.

Most of the biological processes in living cells hardly occur without protein interactions mediated by scaffold proteins. PDZK1 (PDZ domain containing 1) is a scaffold protein containing four PDZ protein interaction domains which interacts with a number of membrane transporter proteins, including ion channels and drug transport proteins, affecting their subcellular signaling transduction, membrane localization, and transport activities [10]. Previous reports showed an interaction between PDZK1 and MRP2 in apical region of HepG2 cells and an interaction between PDZK1 and BCRP in intestinal apical brush-border membrane using pull-down studies [11, 12]. And then these interactions with PDZK1 are involved in regulation of the localization and transport activities of BCRP and MRP2 [11–13]. In view of the regulatory abilities of PDZK1 and the regulation of CBS on BCRP and MRP2, we speculated that PDZK1 might participate in CBS-regulated bile salt delivering and play an important role in restoring biliary transport function in EE-induced intrahepatic cholestasis.

In this study, we used baicalin and mitoxantrone, the common substrates of MRP2 and BCRP respectively, to investigate the regulatory effects of CBS on bile salt transporters in EE-induced intrahepatic cholestasis rats. At the same time, the expression of PDZK1 in the liver of intrahepatic cholestasis rats was also measured to preliminarily explore its role in biliary transport.

## 2. Materials and Methods

### 2.1. Chemicals and Materials

EE and mitoxantrone were obtained from Sigma-Aldrich (St. Louis, MO, USA). Propylene glycol was purchased from Sinopharm Chemical Reagent Co., Ltd. (Shanghai, China). Antibodies were purchased from Santa Cruz Biotechnology Inc. (California, USA). The solvents used for HPLC were of HPLC grade.

CBS (lot number: 140603) was gifted from Wuhan Jianmin Dapeng Pharmaceuticals Co., Ltd. (Wuhan, China). Baicalin was obtained from the National Institute for the Control of Pharmaceutical and Biological Products (Beijing, China). Voucher specimens were deposited at Herbarium of Department of Pharmacy, Tongji Hospital, Tongji Medical College, Huazhong University of Science and Technology, China.

### 2.2. Animals and Treatments

Male Wistar rats weighing 220 ± 20 g were used and kept under laboratory conditions of temperature (25 ± 2°C) and lighting (12:12 h light/dark cycle) with free access to standard laboratory chow and tap water. All rats were acclimatized for one week before experiment. This study was carried out in strict accordance with the guideline of* the National Institutes of Health Guide for the Care and Use of Laboratory Animals*. The protocol was approved by the Ethical Committee on* Animal Experimentation of Tongji Medical College*,* Huazhong University of Science and Technology*,* China.*

Intrahepatic cholestasis rat model was induced by subcutaneous injection of EE (5 mg/kg) for five days (days 1–5). Concurrent treatment of CBS (50 or 150 mg/kg dissolved in 0.5% sodium carboxyl methyl cellulose (CMC-Na)) was given orally once per day for five consecutive days during modeling. Other groups were given equal volumes of vehicles (0.5% CMC-Na). Rats were fasted for 12 h after the last dose of agents before they were anesthetized with sodium pentobarbital (50 mg/kg intraperitoneal injection) and surgical procedures were performed on the sixth day. Some rats were killed by cervical dislocation, from which the livers were removed to analyze mRNA and protein expression levels of PDZK1. Biliary trees of other rats were exposed through midline abdominal incisions. After bile ducts were cannulated and bile was collected for 10 min (equilibrium), the rats were injected with baicalin (20 mg/kg, 0.25 mL/100 g) or mitoxantrone (2 mg/kg, 0.25 mL/100 g) through the caudal vein. Then bile was collected in six 20-minute intervals (0–20, 20–40, 40–60, 60–80, 80–100, and 100–120 min). Biliary secretions of baicalin and mitoxantrone were determined by HPLC.

### 2.3. HPLC Analyses of Baicalin and Mitoxantrone in Rat Bile

HPLC analyses of baicalin and mitoxantrone in rat bile were conducted according to the methods described previously [14–16]. Both chromatographic analyses were performed on a WATERS 2690 Separations Modules HPLC system (Waters, USA) by using a reverse-phase column (Elite Hypersil ODS2 C18 column, 200 × 4.6 mm, ID, 5 *μ*m particle size, Dalian) connected with a guard column (Security Guard, 4.0 × 3.0 mm, Phenomenex).

Peaks of the chromatograms were evaluated with Empower Pro Chromatography Data Software (Waters, USA). The biliary excretion rate and cumulative biliary excretion were calculated by the bile flow and concentration of baicalin or mitoxantrone.

### 2.4. Quantitative RT-PCR

Reverse transcription-quantitative real-time PCR (RT-qPCR) was used to determine mRNA expression of PDZK1 in the liver. Total RNA was extracted from liver samples using TRIzol (Invitrogen, Carlsbad, CA) according to the manufacturer's instructions. PCR primers for PDZK1 and *β*-actin contained the following sequences: PDZK1, 5′-tggaaatgattagaaacggtgg-3′ and 5′-gtcatagggttcatcttgcacatt-3′; *β*-actin, 5′-cgttgacatccgtaaagacctc-3′ and 5′-taggagccagggcagtaatct-3′. The amplified product sizes by each pair of primers were 398 and 110 bp for PDZK1 and *β*-actin, respectively. The mRNA expression levels were normalized to that of *β*-actin. Quantification of the target cDNAs in samples was normalized to the geometric mean of three reference genes (Ct_target_ − Ct_reference  genes_ = ΔCt), and the difference between expression of each target cDNA in the treated groups was expressed to the amount of the control group (ΔCt_treated_ − ΔCt_control_ = ΔΔCt). Fold changes in target gene expression were determined by taking 2 into the power of this number (2^−ΔΔCt^).

### 2.5. Western Blot Analysis

Membrane protein of liver samples was extracted with membrane protein extraction kit (Beyotime Institute of Biotechnology, China) according to the protocol. Protein concentrations were measured with a modified BCA technique. An equal amount of membrane protein (100 *μ*g) per lane was separated with 10% sodium dodecyl sulfate-polyacrylamide gel electrophoresis. After electrophoresis, the gels were transferred to polyvinylidene difluoride membranes which were blocked with Tris-buffered saline containing 5% nonfat milk. Then the membranes were incubated overnight at 4°C in Tris-buffered saline containing 0.1% Tween 20 (TBST), 5% nonfat milk, and anti-PDZK1 (at the dilution of 1 : 200). After being washed three times in TBST, the membranes were incubated with HRP-conjugated secondary antibody for 2 h at room temperature and subsequently processed for enhanced chemiluminescence (ECL) detection using potent ECL kit (Multisciences, China). Signals were detected using a chemiluminescence detection system (IS4000MM Pro, Kodak, USA). *β*-Actin was used as a loading control.

### 2.6. Immunohistochemistry Detection of PDZK1

Livers from rats were perfused with phosphate-buffered saline (PBS), sliced, and fixed with 4% paraformaldehyde. The liver samples were incubated overnight in PBS with 6.8% sucrose, dehydrated with acetone, and embedded. Before staining, semithin sections were incubated for 5 min at 37°C. Subsequently, the sections were incubated for 5 h at 37°C with PDZK1 antibody and then treated with corresponding secondary antibody, followed by incubation with peroxidase–antiperoxidase for 1 h at 37°C. At last, the immunolabeling was visualized by incubation with 3,3′-diaminobenzidine-H_2_O_2_ medium for 10 min at room temperature. Images were captured with an OLYMPUS photomicroscope with digital camera.

### 2.7. Statistical Analysis

All the results were expressed as mean ± SD. Data analyses were performed using SigmaPlot 12.0 software (Systat Software Inc., USA). Statistical analysis was conducted using one-way analysis of variance (ANOVA) and LSD post hoc test. *P* < 0.05 was considered to indicate a statistically significant difference.

## 3. Results

### 3.1. Effect of CBS on Biliary Excretions of Baicalin and Mitoxantrone

**Figure 1:**
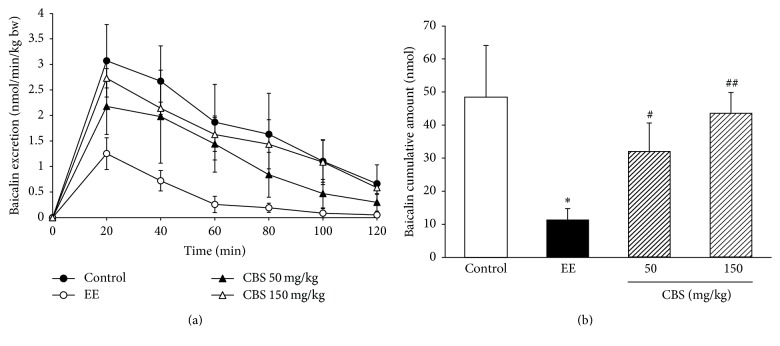
Effect of CBS on biliary excretion of baicalin (*n* = 6). (a) Biliary excretion rate of baicalin. (b) Cumulative biliary excretion of baicalin in two hours. Data are represented as mean ± SD of six rats per group. ^*∗*^*P* < 0.05 versus control group; ^#^*P* < 0.05, ^##^*P* < 0.01 versus EE group by one-way ANOVA and LSD post hoc test.

Baicalin is a major bioactive component of* Scutellaria baicalensis*, the hepatic excretion of which is dominantly controlled by MRP2 [14, 17]. To evaluate the transport ability of MRP2, we detected the bile excretion of baicalin in EE-induced intrahepatic cholestasis rats. After baicalin was injected intravenously, bile was collected consecutively and the concentration of baicalin therein was measured by HPLC. The excretion rate and cumulative biliary excretion of baicalin are shown in Figures 1(a) and 1(b). EE apparently decreased the cumulative biliary excretion of baicalin compared with that in the control group (*P* < 0.05). The cumulative biliary excretion of baicalin was significantly higher in CBS (50 and 150 mg/kg) treated groups than that in the EE group (*P* < 0.05 and *P* < 0.01).

**Figure 2:**
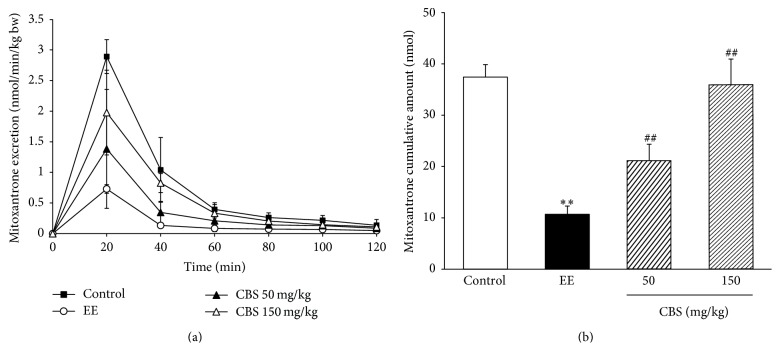
Effect of CBS on biliary excretion of mitoxantrone (*n* = 6). (a) Biliary excretion rate of mitoxantrone. (b) Cumulative biliary excretion of mitoxantrone in two hours. Data are represented as mean ± SD of six rats per group. ^*∗∗*^*P* < 0.01 versus control group; ^##^*P* < 0.01 versus EE group by one-way ANOVA and LSD post hoc test.

Mitoxantrone is a specific substrate of BCRP [18], and changes in the expression and transport activity of BCRP in the liver directly affect the biliary excretion of mitoxantrone [16]. The effects of CBS on the excretion rate and cumulative biliary excretion of mitoxantrone were also evaluated in intrahepatic cholestasis rats. As shown in Figures 2(a) and 2(b), EE significantly reduces the cumulative biliary excretion of mitoxantrone (*P* < 0.01). Compared with the EE group, CBS (50 mg/kg and 150 mg/kg) greatly reversed EE-induced decrease in the cumulative biliary excretion (*P* < 0.01 and *P* < 0.01). Thus, CBS was able to restore the biliary transport functions of MRP2 and BCRP in a dose-dependent manner.

### 3.2. RT-qPCR, Western Blot, and Immunohistochemistry Analysis of PDZK1

**Figure 3:**
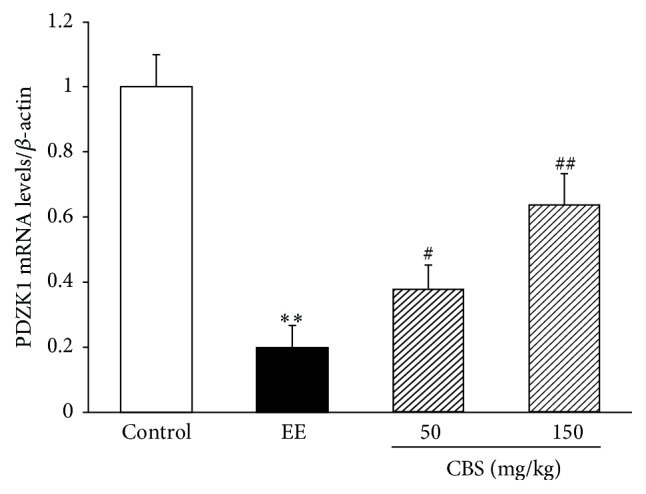
mRNA levels of PDZK1, measured by RT-PCR and normalized to *β*-actin mRNA, relative to the control set as 1. Data are represented as mean ± SD of six rats per group. ^*∗∗*^*P* < 0.01 versus normal group; ^#^*P* < 0.05, ^##^*P* < 0.01 versus EE group by one-way ANOVA and LSD post hoc test.

PDZK1 is highly expressed in the liver where it links receptors, ion channels, transporters, and cytosolic components [19]. It has been well-demonstrated that PDZK1 can interact with some bile transporters and might play an important role in biliary excretion. In this study, we analyzed the possible modulation of PDZK1 in EE-induced intrahepatic cholestasis rats and subsequently explored the potential function of CBS in PDZK1 expression to clarify the choleretic mechanism. The mRNA expression of PDZK1, as detected by RT-qPCR, significantly decreased in EE-induced intrahepatic cholestasis rats compared with that in normal rats. Administrating rats with CBS (50 or 150 mg/kg) notably increased PDZK1 mRNA expression compared with that of EE-induced intrahepatic cholestasis rats (Figure 3).

**Figure 4:**
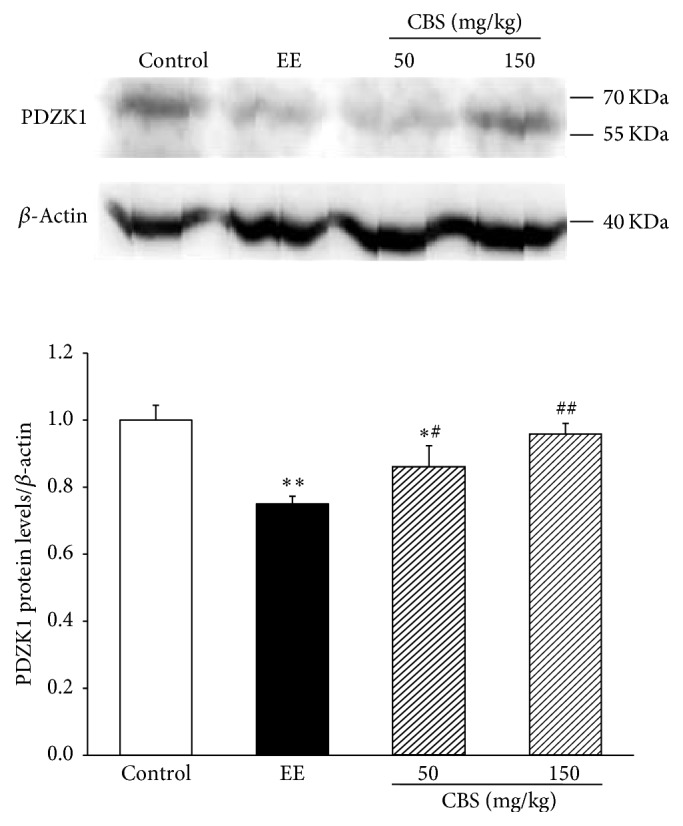
Protein levels of PDZK1, measured by western blot and normalized to *β*-actin protein, relative to the control set as 1. Data are represented as mean ± SD of six rats per group. ^*∗*^*P* < 0.05, ^*∗∗*^*P* < 0.01 versus normal group; ^#^*P* < 0.05, ^##^*P* < 0.01 versus EE group by one-way ANOVA and LSD post hoc test.

**Figure 5:**
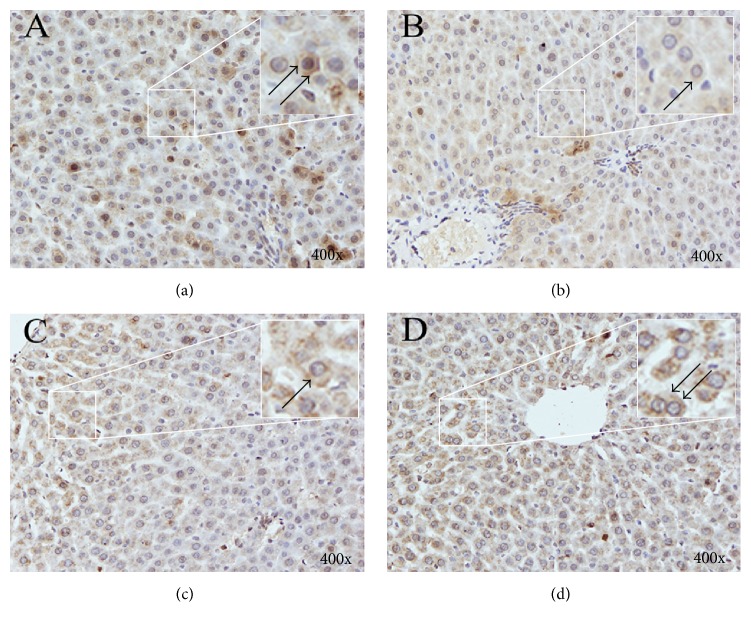
Immunohistochemistry for PDZK1 expression in rat livers. (a) Control group. (b) EE group. (c) 50 mg/kg CBS group. (d) 150 mg/kg CBS group. The upper right images of each figure are enlargements of the square areas indicating the partial drawing of PDZK1 on the hepatocytes. Positive staining (brown staining) products are denoted by black arrows. Magnification: ×400.

The effect of CBS on the protein expression of PDZK1 is exhibited in Figure 4. EE evidently reduced the protein level of PDZK1 compared with that of the control group, which was markedly reversed through coadministration with CBS (50 or 150 mg/kg). The immunohistochemical results were consistent with those of quantitative Western blot analysis. PDZK1 in the livers of normal rats was predominantly distributed on the membrane of hepatocytes. PDZK1 staining was significantly attenuated in the EE group, which was remarkably reinforced by treatment of CBS (50 or 150 mg/kg) (Figure 5). Thus, CBS was involved in recovering PDZK1 expression in EE-induced intrahepatic cholestasis rats.

## 4. Discussion

CBS has already been recorded in the Pharmacopoeia of the People's Republic of China and established the corresponding quality control standards [20]. Based on previous studies, we used bile flow experiment to elucidate the mechanism by which CBS protected rats from EE-induced intrahepatic cholestasis. CBS significantly improved the biliary transport functions of MRP2 and BCRP in such rats and upregulated the mRNA and protein expression levels of PDZK1.

MRP2 and BCRP (also known as ABCC2 and ABCG2, resp.), as membrane transporter members of the ATP-binding cassette (ABC) family, are highly expressed on the apical membrane of hepatocytes [21, 22]. In general, MRP2 and BCRP are efflux transporters responsible for the transport of bilirubin, glucuronidated- and sulfated-bile acids, and several drugs from hepatocytes to biliary duct [23]. Under pathological circumstances of cholestasis, accumulation of potentially toxic compounds is accompanied by decreased expressions of MRP2 and BCRP [24, 25]. Our previous studies, however, showed that the mRNA and protein expressions of MRP2 and BCRP were increased by CBS treatment in EE-induced intrahepatic cholestasis rats [9]. Therefore, to further validate the influence of CBS on their transport function, we observed the biliary excretion and cumulative biliary excretion of baicalin and mitoxantrone, respectively, finding that the transport activities of MRP2 and BCRP were obviously enhanced by CBS in a dose-dependent manner. Hence, CBS treatment mitigated EE-induced intrahepatic cholestasis, at least partially by boosting the functions of MRP2 and BCRP to decrease the accumulation of toxic compounds in the liver.

As an ideal substitute for* Calculus Bovis*, CBS has been widely used in clinical and medical preparations. Although some bile acids have been identified as the most important bioactive constituents in CBS, the actual pharmacologically active components have not been determined yet [7]. It is now well-documented that some of these bile acids in CBS are capable of improving bile transport function in intrahepatic cholestasis. Liu and others showed that cholic acid and deoxycholic acid increased the mRNA expression of MRP2 in primary human hepatocytes [26]. Azzaroli and others reported that ursodeoxycholic acid upregulated the expression of placental BCRP in patients with intrahepatic cholestasis of pregnancy [27], and we have previously verified that ursodeoxycholic acid augmented bile efflux of baicalin from the liver [14]. Moreover, Gerk and others demonstrated that ursodeoxycholic acid, tauroursodeoxycholic acid, and glycoursodeoxycholic acid stimulated MRP2-mediated transport of *β*-estradiol 3-(*β*-D-glucuronide) and *β*-estradiol 17-(*β*-D-glucuronide), two substrates of MRP2 [28]. These all suggest that certain constituents of bile acids in CBS can regulate these bile salt transporters. However, the regulatory mechanism of CBS on the functions of bile salt transporters remains unknown.

PDZK1, a scaffold protein, contains four PDZ-banding domains generally comprising 80–90 amino acids, which play crucial roles in targeting proteins to specific cell membranes, assembling proteins into signaling complexes for efficient transduction, and regulating the functions of transmembrane proteins [10]. Yeast two-hybrid and pull-down studies using recombinant proteins have identified a number of membrane transporters interacting with PDZK1, such as OATP1A, OCTN2, and PEPT2, including MRP2 and BCRP [10]. Interaction of PDZK1 with various membrane transport proteins occurs at extreme C-terminal PDZ binding motif which is in a class I site defined by amino sequence ([S/T]-X-[Φ]), where Φ is hydrophobic amino acids and X is any amino acids [12]. The C-terminus of MRP2 containing PDZ domain motif, as evidenced by affinity pull-down assays, binds the fourth PDZ domain of PDZK1. Removal of this PDZ-binding motif significantly reduces normal apical localization of MRP2 in polarized HepG2 cells [11]. Greater interests, however, rose because the C-terminal sequence of BCRP is K-Y-S in both humans and mice and does not match PDZ binding motif and pull-down assays showed that PDZK1 can interact with BCRP. So it is possible that the interaction between PDZK1 and BCRP is different from MRP2 [12]. But Shimizu and others demonstrated that PDZK1 regulates BCRP by increasing its expression level and transcellular transport function in transfected MDCKII/BCRP/PDZK1 cells, and much less BCRP was expressed and localized on the apical membranes in* pdzk1* (−/−) mice than that in wild-type mice [12]. In short, PDZK1 exerts fundamental effects on the apical localization of these two membrane transporters. However, the information about the localization of PDZK1 at the apical membrane of hepatocytes in vivo is still limited [10]. Further in vitro and in vivo studies focused on the apical localization of PDZK1 and its interactions with MRP2 and BCRP in liver are still necessary.

Hence, we postulated that CBS relieved intrahepatic cholestasis by enhancing the functions of MRP2 and BCRP via the PDZK1 pathway. Since CBS upregulated the mRNA and protein expression levels of PDZK1 in EE-induced intrahepatic cholestasis rats measured by RT-PCR, western blot, and immunohistochemistry analysis, the bile transport abilities of MRP2 and BCRP were recovered, respectively. Conclusions can be drawn that this process mediated by CBS may be via the PDZK1 pathway.

## 5. Conclusions

The results demonstrated that CBS enhances the activities of MRP2 and BCRP and simultaneously upregulates the expression of PDZK1. The possible regulations of PDZK1 on the localization and function of MRP2 and BCRP may play an importance role in restoring the biliary transport function of EE-induced intrahepatic cholestasis rats. Investigations on the detailed relationships between the choleretic mechanisms and PDZK1 regulated hepatic efflux transporters are still ongoing.
